# Complete Genome Sequences of Two Nosocomiicoccus ampullae Strains and a Growth-Adapted Mutant

**DOI:** 10.1128/MRA.00747-21

**Published:** 2021-12-16

**Authors:** Christopher F. Schuster, Frank Sommer, Birgit Strommenger, Guido Werner, Franziska Layer

**Affiliations:** a Division of Nosocomial Pathogens and Antibiotic Resistance, Robert Koch Institute, Wernigerode, Germany; b Universitätsklinikum Gießen und Marburg GmbH, Institut für Medizinische Mikrobiologie und Krankenhaushygiene, Marburg, Germany; Indiana University, Bloomington

## Abstract

Here, we present the circular and complete genome sequences of the Nosocomiicoccus ampullae isolate 19-00310 and type strain DSM 19163. To our knowledge, these represent the first complete, circular chromosomes in the entire genus. Sequencing of a growth-adapted mutant suggests iron availability as a factor for growth improvement.

## ANNOUNCEMENT

Nosocomiicoccus ampullae is a Gram-positive, halophilic, aerobic, nonmotile coccal bacterium (1) and a member of the *Staphylococcaceae* family (2). Apart from nosocomial association, not much is known about the occurrence, habitat, and pathogenicity of this species. In fact, isolation is rare, with PubMed currently listing only two publications (1, 3) and two (noncontiguous) assemblies putatively assigned to this species.

**FIG 1:**
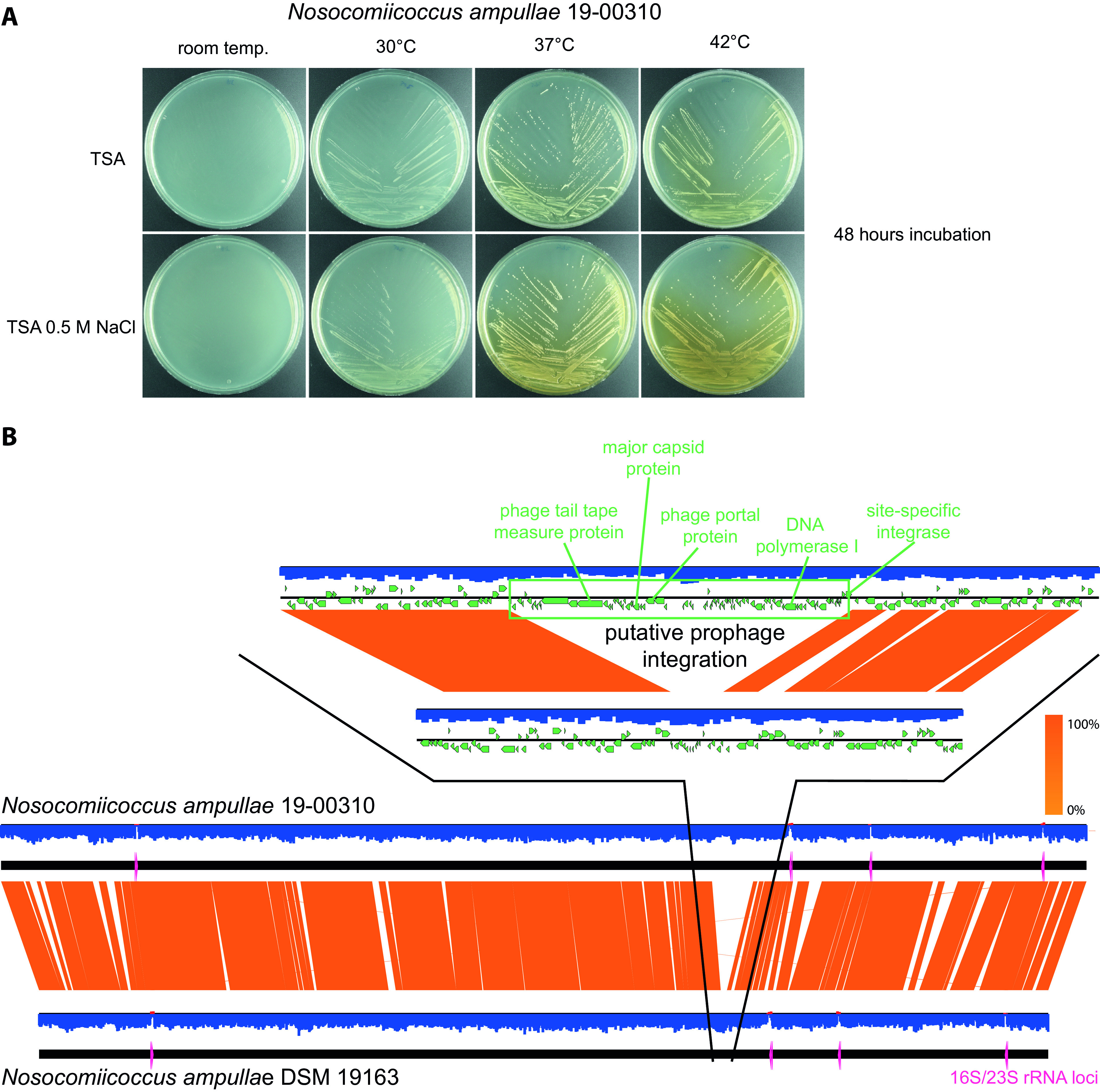
(A) Growth of Nosocomiicoccus ampullae strain 19-00310 on TSA at different temperatures with and without the addition of 0.5 M NaCl. When the strain was cultivated at 37°C or 42°C, a diffused pigment is visible in the agar. (B) Genome alignment of strains 19-00310 and DSM 19163 is shown at the bottom (made with Easyfig v2.2.5 [12]). The insertion of a putative prophage in strain 19-00310 is shown at the top. G+C content is shaded in blue (GC/GCAT < 0.5)/red (GC/GCAT > 0.5), 16S/23S rRNAs are highlighted in pink, BLASTN homology between strains is shaded in orange, and genes are depicted as green arrows.

Isolate 19-00310 was collected in 2018 in a German hospital during routine diagnostic analysis of a joint lesion. Joint aspirate was streaked on Columbia (5% sheep blood) agar, chocolate agar, and Schaedler agar (Becton, Dickinson) and also was used to inoculate thioglycolate broth (Oxoid). There was no sign of bacterial growth on days 1, 2, and 7 at 36°C. On day 13, the thioglycolate broth was streaked on Columbia agar and Schaedler agar, and small colonies appeared after 24 h. Matrix-assisted laser desorption ionization–time of flight mass spectrometry (MALDI-TOF MS) (Bruker) identification was inconclusive; therefore, the isolate was sent to the Robert Koch Institute (RKI) for further investigation. The isolate grew at 30°C to 42°C on tryptic soy agar (TSA) (BD) plates but showed the best growth at 37°C with 0.5 M NaCl added. Growth was also improved on marine medium 2216 (HiMedia). After 48 h, colonies reached sizes of 0.4 to 0.8 mm on TSA, and the diffusion of a brown metabolite was evident (Fig. 1A). To improve growth on TSA, isolate 19-00310 was continuously passed over to fresh TSA plates 20 times, resulting in strain 19-00310-R20. Strain DSM 19163 was acquired as a freeze-dried vial from the German Collection of Microorganisms and Cell Cultures (DSMZ) and was grown on TSA and solid marine medium.

DNA of all strains was prepared from cells that had been grown for 3 to 4 days at 37°C on solid marine medium, using the DNeasy blood and tissue kit (Qiagen) for Illumina sequencing and the MagAttract high-molecular-weight (HMW) kit (Qiagen) with an SPRISelect (Beckman Coulter) size selection step for MinION sequencing (Oxford Nanopore Technologies [ONT]). Short-read sequencing was performed for all strains and was conducted on a MiSeq system (Illumina) in paired-end mode for 600 cycles using the Nextera XT kit (Illumina) for library preparation. Long-read sequencing of strains 19-00310 and DSM 19163 was performed on a MinION system (ONT) with intact HMW DNA using the rapid adapter SQK-RAD004 kit (ONT) and FLO-MIN106 flow cells (ONT), with sequencing time of 16 h (19-00310) or 12.75 h (DSM 19163). Long-read base calling was done with model dna_r9.4.1_450bps using ONT Guppy v4.4.2+9623c16 and v4.0.15+5694074 and was quality controlled using NanoPlot v1.20.0 with fastq files (4).

Short reads were adapter clipped using Trimmomatic v0.36+dfsg-1 (5). Long reads were clipped with Porechop v0.2.4 (https://github.com/rrwick/Porechop) and filtered with Filtlong v0.2.0 (https://github.com/rrwick/Filtlong) (best 100 Mbp). *De novo* hybrid assemblies were created for strains 19-00310 and DSM 19163 using Unicycler v0.4.9b (6) with SPAdes v3.13.0 (7) and resulted in one circular chromosome for each strain and a small, circular plasmid for strain DSM 19163. The genomes were rotated in Unicycler using the N. ampullae *dnaA* gene and annotated using the NCBI Prokaryotic Genome Annotation Pipeline (8). Default parameters were used for all software except where otherwise noted. Sequencing and assembly statistics are listed in Table 1. Strain 19-00310 showed a fastANI (9) similarity score of 97.62% with respect to the hybrid assembly of the type strain N. ampullae DSM 19163 and contained a putative novel prophage (Fig. 1B). The second proposed N. ampullae strain (3) from GenBank (GenBank assembly number GCA_001696685.1) showed fastANI scores of 82.41% and 95.64% with respect to the N. ampullae and Nosocomiicoccus massiliensis (10) type strains, respectively. With a G+C content of 36.2%, we propose that this strain does not belong to N. ampullae (G+C content of 34.5%) but instead belongs to the *N. massiliensis* group (G+C content of 36.5%) but was misassigned due to the lack of next-generation sequencing data at the time of deposit.

**TABLE 1:** Sequencing summary of Nosocomiicoccus ampullae strains

Parameter^*a*^	Finding for:		
	*N. ampullae* DSM 19163	*N. ampullae* 19-00310	*N. ampullae* 19-00310-R20
Year of isolation	2005	2018/2019	2020
Source	DSMZ	Germany, patient	Laboratory derivative of 19-00310
Illumina sequencing
No. of reads	1,159,280	1,254,160	1,634,220
Size (bp)	228,474,683	323,296,777	362,691,171
Avg coverage (×)	147	194	218
SRA accession no.	SRR15012077	SRR15012070	SRR15012079
ONT sequencing			
No. of reads	1,263,774	1,163,633	
Size (bp)	6,659,297,454	8,460,836,675	
Read *N*_50_ (bp)	9,596	12,857	
Median read length (bp)	3,126	4,534	
Avg coverage (×)	4,298	5,077	
SRA accession no.	SRR15012078	SRR15012071	
Assembly			
No. of contigs	2	1	Not assembled
Total genome size (bp)	1,551,959	1,666,268	
Chromosome size (bp)	1,549,333 (complete)	1,666,268 (complete)	
Plasmid size (bp)	2,626 (complete)		
G+C content (%)	34.5	34.3	
Total no. of genes	1,605	1,766	
Total no. of CDSs	1,539	1,700	
No. of coding genes	1,522	1,674	
No. of CDSs with protein	1,522	1,674	
No. of RNA genes	66	66	
No. of rRNAs			
5S	4	4	
16S	4	4	
23S	4	4	
No. of complete rRNAs			
5S	4	4	
16S	4	4	
23S	4	4	
No. of tRNAs	50	50	
No. of noncoding RNAs	4	4	
Total no. of pseudogenes	17	26	
No. of CRISPR arrays	0	1	
Plasmid			
Size (bp)	2,626 (complete)		
Name	pDSM19163_1		
No. of genes	1 (replication initiation)		
Assembly accession no.			
Chromosome	CP079110	CP079109	
Plasmid	CP079111		
BioSample accession no.	SAMN19605268	SAMN19605271	SAMN19981403
BioProject accession no.	PRJNA735953	PRJNA735957	PRJNA735957
fastANI score (%) with respect to:			
N. ampullae DSM 19163 (GenBank accession no. GCF_014202595.1)	99.98	97.62	
N. ampullae LUREC (GenBank accession no. GCF_001696685.1)	82.39	82.65	
SNP			
Position			269757
Observed base			G>T (T:217 G:0)
Change (nucleotide/amino acid)			c.613G>T/p.Asp205Tyr
Locus and/or CDS			ABC transporter substrate-binding protein (siderophore receptor)
Position			782217
Observed base			C>T (T:76 C:0)
Change (nucleotide/amino acid)			c.919C>T/p.Leu307Leu (silent)
Locus and/or CDS			IS*3* family transposase
Position			1040644
Observed base			C>A (A:114 C:0)
Change (nucleotide/amino acid)			
Locus and/or CDS			Intergenic
Position			1344421
Observed base			C>A (A:113 C:0)
Change (nucleotide/amino acid)			
Locus and/or CDS			RBS of *rsbV*

a CDS, coding sequence; RBS, ribosomal binding site.

Single-nucleotide polymorphisms (SNPs) in strain 19-00310-R20 were identified with Snippy v4.4.5 (11) using the 19-00310 assembly as a reference. Analysis revealed (among other mutations) an amino acid exchange in an ABC transporter gene that is putatively involved in iron transport (Table 1). This could suggest that iron availability is limiting for N. ampullae on TSA, and this is further supported by improved growth on marine medium, in which ferric citrate is supplemented.

### Data availability.

Illumina and MinION data have been deposited under BioProject accession numbers PRJNA735953 (SRA accession numbers SRR15012077 and SRR15012078) and PRJNA735957 (SRA accession numbers SRR15012070, SRR15012071, and SRR15012079). The assemblies can be found in GenBank under accession numbers CP079110 (Nosocomiicoccus ampullae DSM 19163), CP079111 (plasmid pDSM19163_1), and CP079109 (Nosocomiicoccus ampullae 19-00310). Strains 19-00310 and 19-00310-R20 can be acquired from the authors, and strain DSM 19163 can be acquired from the DSMZ.
